# Mental Rotation Performance in Male Soccer Players

**DOI:** 10.1371/journal.pone.0048620

**Published:** 2012-10-30

**Authors:** Petra Jansen, Jennifer Lehmann, Jessica Van Doren

**Affiliations:** Institute of Sport Science, University of Regensburg, Regensburg, Bavaria, Germany; University Medical Center Groningen UMCG, The Netherlands

## Abstract

It is the main goal of this study to investigate the visual-spatial cognition in male soccer players. Forty males (20 soccer players and 20 non-athletes) solved a chronometric mental rotation task with both cubed and embodied figures (human figures, body postures). The results confirm previous results that all participants had a lower mental rotation speed for cube figures compared to embodied figures and a higher error rate for cube figures, but only at angular disparities greater than 90°. It is a new finding that soccer–players showed a faster reaction time for embodied stimuli. Because rotation speed did not differ between soccer-players and non-athletes this finding cannot be attributed to the mental rotation process itself but instead to differences in one of the following processes which are involved in a mental rotation task: the encoding process, the maintanence of readiness, or the motor process. The results are discussed against the background of the influence on longterm physical activity on mental rotation and the context of embodied cognition.

## Introduction

It is the main goal of this study to investigate the visual-spatial cognition in male soccer players, which has not been investigated until now. Up to this point studies exist which are concerned with the perceptual-cognitive skills of sports-experts, for example attentional skills [1], visual-search behavior [2] or memory performance [3]. These are all cognitive skills which are relevant to sports performance and are discussed in the framework of expertise research. In a new study, general cognitive functions, namely executive functions, were investigated in High Division soccer players (HD) compared to Low Division players (LD) and a standardized norm group [4]. They showed that both groups of soccer players had a better performance in the measurements of the executive functions than the norm group, and that the performance of the HD players was better than that of the LD players. Quite interestingly the performance in the executive function test correlates with the number of goals and assists each player had two seasons later. One important aspect of executive function is the aspect of working memory. Because we know that working memory plays an important role in other cognitive abilities, for example visual-spatial cognitive abilities [5], it might be assumnd that soccer players who showed higher executive functions performance also have enhanced visual-spatial abilities. According to a meta-analysis of Linn and Peterson, visual spatial abilities can be differentiated into the abilities of visualization, orientation, and mental rotation [6]. Currently mental rotation is the best-investigated component of these visual-spatial abilities. Mental rotation describes the ability and the process of imagining how an object appears if it is rotated from its original position [7]. This ability is well investigated in several fields including the study of gender differences [8], developmental psychology [9], neuroscience [10], and general psychology [11], [12]. During mental rotation several processing stages occur [13], [14]: (1) perceptual processing, (2) identification and discrimination of stimuli, (3) identification of orientation, (4) mental rotation, (5) judgment of parity, (6) response selection, and (7) execution. Whereas the first three stages are perceptual stages, stages 4 and 5 comprise the rotation process itself. The last two stages are two stages of the decision process. Stages 4 and 5 entail working memory processes as a part of executive functions.

In experimental psychology the relationship between mental rotation and motor processes has been questioned. This relationship was investigated by the work of Wexler, Kosslyn, and Berthoz [15] who assumed that the mental rotation process was a covert motor rotation and the work of Wohlschläger and Wohlschläger [16] which showed that motor and mental rotation share the same processes. In the study of Wexler et al. [15] participants performed manual and mental rotation simultaneously: They had to rotate a joystick while they completed mental rotation tasks. When the mental and manual rotation tasks differed in direction of rotation, reaction time was slower than for rotations in the same direction.

In addition to these studies there are several other studies which did not specifically investigate the effect of motor processes itself, but focused on the effect of long term or short term physical activity on mental rotation. These physical activity studies have to be distinguished concerning the kind of mental rotation tasks they used: object-based transformation tasks vs. (egocentric) perspective transformation tasks. In an object-based transformation task the mental rotation is conducted according to a stationary environment and viewer perspective as an object changes. This is in contast to an (egocentric) perspective transformation where the object and environment remain the same while the viewer's frame of reference changes [17]. The use of abstract objects requires an object transformation whereas the use of body pictures as stimuli material can induce either an object or a perspective transformation. The use of an object- versus perspective transformation with body figures as stimuli depends on the kind of judgment: A same-different decision requires an object transformation whereas a left-right judgment, for some parts of the body figures, can induce a perspective transformation [18].

Studies with object based transformations which investigate the effects of long-term physical or musical activity on mental rotation [19] using a psychometric mental rotation test [20] have found a better mental rotation performance for sports and music students compared to students of education science. This effect was more specifically defined in another study, in which two groups of university students received 10-months of wrestling or running training [21]. While the pretest data of all the participants were comparable, the posttest showed that the wrestling students outperformed the running students. This result suggests that training which includes highly coordinative and rotational movement aspects improves mental rotation performance better than training which do not include these aspects. This is in line with a study showing a positive influence of juggling on mental rotation performance [22]. In another study the long term effect of physical activity with rotational movements was investigated for object-based and egocentric perspective mental rotation tasks. It was shown that experts for rotational movements had a better performance than non-experts if they only had to decide if the left or right arm of people in pictures was raised (perspective transformation) but not for object based transformations, experiment 1 of their study [18]. This result is contradictory to the studies mentioned above showing the relationship between physical activity and the object based mental rotation tasks [21], [19], [22]. One reason for this might be that the stimuli used in the study of Steggemann et al. [18] are not comparable to the stimuli in the studies mentioned above. Furthermore, the stimuli in experiment 1 of their study (letters vs body postures) are not comparable to each other, because the human body postures are more complex than the letters in terms of their components and surface description. Even though they mention that there are studies showing that the complexity of the objects did not influence the mental rotation process [23] other studies contradict this idea and show that the number of bends, cubes, and configurations modulate the mental rotation rate [24].

Therefore, we used objects in this study, which are comparable to the objects in “positive” effect studies of long term specific physical activity on mental rotation, as well as comparable to each other. Steggemann et al. [18] also investigated the influence of rotational expertise in participants whose athletic background requires a highly body awareness, specifically artistic or wheel gymnastics. These activities imply a body centered or egocentric perspective. An object based transformation or object mental rotation task requires the perception of space from an outside stationary point of view, that means a third person view or an object centered view (allocentric or exocentric). Due to these differences in transformation strategies we chose to investigate the object based mental rotation performance of soccer players, who train by perceiving objects and analyzing spatial relationships from a non-centered point of view. Because of their better visual-search behavior [1] and better executive functioning [4] we hypothesize that soccer-players show a better mental rotation performance in object-based transformations and that this advantage might be pronounced with embodied stimuli. Due to the well-known gender differences in mental rotation [25], [8] only males participated in this study.

## Methods

### Participants

Forty males, 38 University students and 2 university employees (one with vocational training and one with a university degree) (mean age 24.48 years, *SD* = 3.41) participated in this study. Twenty males (mean age 23.7 years, *SD*  = 1.48) were soccer players, and 20 males were non-athletes (mean age 25.25 years, *SD*  = 4.56). The mean number of hours per week practicing soccer was *M* = 5.98 (*SD*  = 1.42) for the soccer players and *M* = 0 (*SD*  = 0) hours for the non-athletes, which differs significantly, *F*(1,38)  = 350.21, *p*<.001, *η2* = .90. The Body-Mass Index (kg/m^2^) did not differ between each group, *F*(1, 38) = .42, *n.s.* (soccer players, *M* = 22.63, *SD*  = 1.31; non-athletes, *M* = 23.20, *SD*  = 3.72). All participants gave their written informed consent to participate in this study. The institutional review board confirmed that a review from the institional review board is not necessary for this study.

### Material

**Figure 1:**
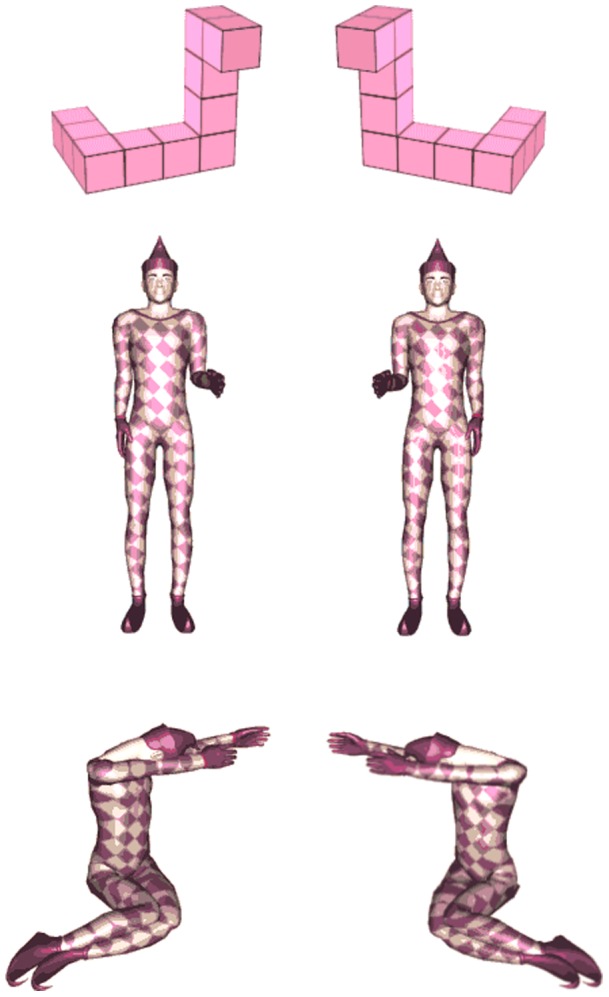
Sample items of the chronometrical mental rotations tasks (cube figures, human figures, body postures).

A computer mental rotation test with three different stimulus types, one abstract figure (cubes figures) and two embodied figures (human figures, and body postures), was solved by all participants. The three stimulus types were presented using presentation software on a laptop with a 17-inch display. The “cube” stimulus types consisted of pink drawings of six different cube figures similar to “classical stimuli figures” [7]. The “human figures” stimulus types consisted of six different human figures. The “body postures” stimulus types consisted of six different figures. An example of each stimulus type is given in Figure 1 [17]. Three images of the particular stimulus type were used as original figures and three mirror figures were constructed from these original figures. In each stimulus type the two stimuli were presented pairwise with an angular disparity of 0°, 30°, 60°, 90°, 120°, 150°, or 180°, which was obtained by the rotation of the comparison figure. Half of the trials were pairs of identical images and half of them were mirror-reversed images. All of the stimuli were coloured pink and displayed against a white background. All stimuli were rotated in picture plane (a roll rotation). Maximum size of each stimulus on the display was 5 cm.

### Procedure

All participants were tested separately. A trial was initiated by a fixation cross in the center of a white screen. Participants had to decide as quickly and as accurately as possible if the stimuli presented were either the same or different, which means identical to the comparision stimuli or mirror reversed respectively. “Same” was indicated by pushing the right mouse button; “different” was indicated by pushing the left mouse button. Each trial began with a 500ms white background. Thereafter the pair of stimuli appeared and remained on the screen until the participant responded. All participants received feedback in form of a “+” for a correct answer and a “−” for an incorrect answer. The feedback was presented for 500ms on the screen. The next trial began after 1500 ms.

Each participant performed two blocks of 126 experimental trials: 3 stimulus types (cubes figures vs human figures vs body postures) * 2 trial types (same or different) *7 angular disparities (0°, 30°, 60°, 90°, 120°, 150°, or 180°) * 3 reference stimuli. The order of presentation was the randomized.

### Statistical analysis

The reaction time (RT) data were trimmed for outliers. RTs more than 2 SDs above or below the mean per condition and per subject were excluded. The program SPSS 18.0 was used to analyze the data. Two analyses of variance for the dependent variables “reaction time” and “error rate” were calculated with the between subject factor “group” (soccer player vs non-athletes) and the within-subject factors “angular disparity” (0°, 30°, 60°, 90°, 120°, 150°, 180°) and “stimulus type” (cube figures vs human figures vs body postures). One analysis of variance was calculated with the dependent variable “mental rotation speed” and the between subject factor “group” (soccer players vs non-athletes). During mental rotation several processing stages occur (see introduction): To investigate the group effect on mental rotation itself the mental rotation process needs to be excluded from the other processes. This can be done by analyzing mental rotation speed which indicates the rotation process itself.

Mental rotation speed was calculated as the inverse of the slope of the regression line separately for each subject, relating RT to angular disparity and was expressed as degrees per second. We further analyzed the reaction time dependent on stimulus time and group at an angular disparity of 0°, which corresponds to the intercept of the reaction time function. The intercept of the reaction time function reflects the perceptual comparision stages, and the decision processes in the mental rotation process.

## Results

### Reaction time

**Figure 2:**
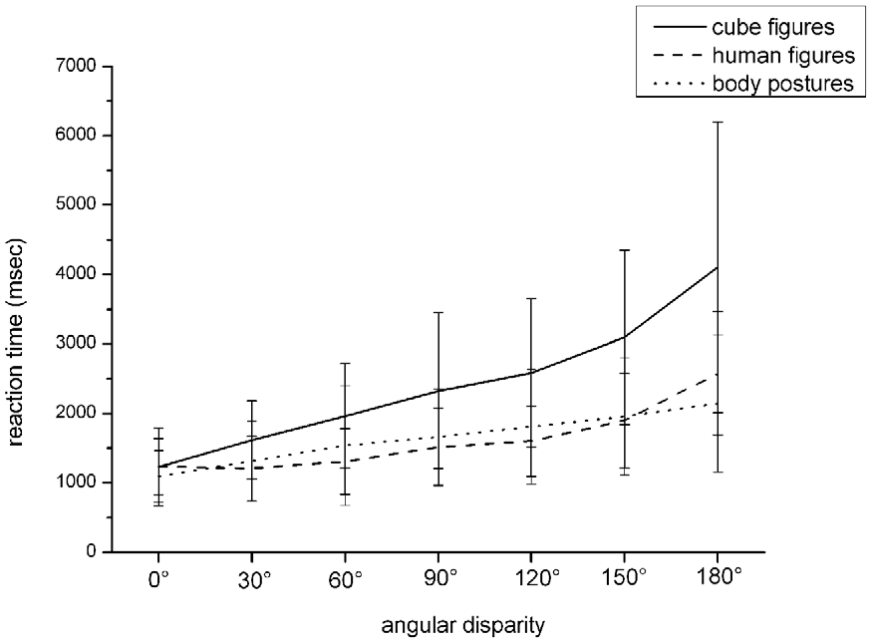
Reaction time dependent on stimulus type and angular disparity (mean, standard deviation).

Concerning reaction time, the analysis of variance showed a main effect for the factors “stimulus type”, *F*(2,76)  = 69.58, *p<*.001, *η^2^* = .647, and for the factor “angular disparity”, *F*(6,228)  = 112.34, *p*<.001, *η^2^* = .747, and a significant interaction between both factors, *F*(12,456)  = 21.12, *p*<.001, *η^2^* = .357 (see Figure 2). Figure 2 shows that the reaction time for cube figures was higher than the reaction time for body posture figures which was higher than that of the human figures for those which had an angular disparity of 60°, 90°, 120°, and 180° (all contrasts p<.05). At an angular disparity of 0°, the reaction time for body postures was faster than that of the cube figures, *F*(1,39)  = 7.01, p<.05, *η^2^* = .15 and human figures, *F*(1,39)  = 5.87, p<.05, *η^2^* = .13, but did not differ between the latter ones, *F*(1,39)  = 0.0, n.s., *η^2^* = .15. At the angular disparities of 30° and 150°, reaction time was higher for cube figures than for the body postures (30°: *F*(1,39)  = 11.14, *p*<.01, *η^2^* = .222, and 150: *F*(1,39)  = 59.43, *p<*.001, *η^2^* = .604), and human figures (30°: *F*(1,39)  = 32.74, *p*<.001, *η^2^* = .456, and 150°: F(1,39)  = 64.91, *p<*.001, *η^2^* = .625) but did not differ between body postures and human figures, (30°: *F*(1,39)  = 2.04, *n.s.* , and 150°: *F*(1,39)  = .432, *n.s.*).

Furthermore, there was a significant interaction between “stimulus type” and “group”, *F*(2,76)  = 3.728, *p*<.05, *η^2^*.089. Simple interaction-contrasts revealed a significant difference between the correct answers given by soccer-players and non-athletes for the body postures stimuli compared to the cube figure stimuli, *F*(1,38)  = 4.75, *p*<.05, *η^2^* = .11 and an almost significant difference between both groups on reaction time on the human figures compared to cube figures, *F*(1,38)  = 3.76, *p* = .06, *η^2^* = .09 (see table 1). Compared to the reaction time for cube figures, soccer players needed less time to answer in both embodied stimuli condition. There was neither a main effect of the factor “group” nor any other significant interaction.

**Table 1:** Mean reaction time (mean, SD) dependent on group and stimuli type.

	Cube figures	Human postures	Body postures
Non-Athletes	2401.13 (776.36)	1763.51 (509.51)	1810.67 (713.64)
Soccer players	2458.91 (1035.51)	1476.22 (515.73)	1478.67 (627.38)

### Error rate

**Figure 3:**
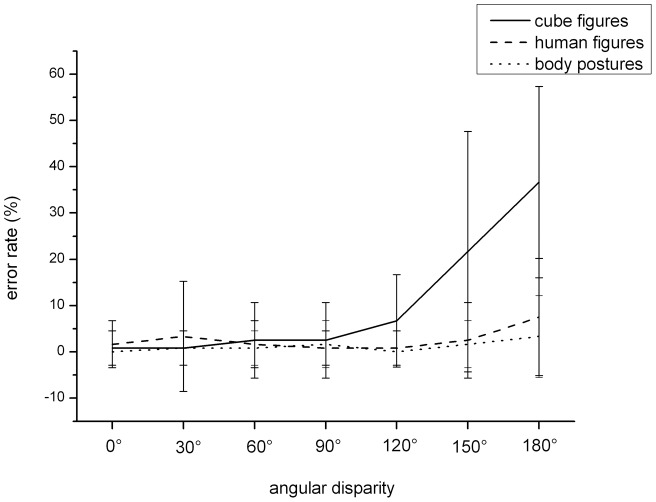
Error rate dependent on stimulus type and angular disparity (mean, standard deviation).

Concerning error rate, the analysis of variance showed a main effect for the factors “stimulus type”, *F*(2,76)  = 55.77, *p*<.001, *η^2^* = .559, and “angular disparity”, *F*(6,228)  = 43.47, *p*<.001, *η^2^* = .543, and a significant interaction between both factors, *F*(12,456)  = 30.67, *p*<.001, *η^2^* = .447 (see Figure 3). Figure 3 shows that there was no difference between the error rate for the cube figures and the two embodied stimulus types at angular disparities of 0°, 30° and 60°, however there was a significant difference for 90°, *F*(2,78)  = 5.49, *p*<.01, *η^2^* = .123, 120°, *F*(2,78)  = 21.54, *p<*.001, *η^2^* = .356, 150°, *F*(2,78)  = 27.35, *p<*.001, *η^2^* = .412, and 180°, *F*(2,78)  = 61.36, *p*<.001, *η^2^* = .611. These differences were due to a higher error rate for cube figures than for body postures and human figures but error rate (all p<.05) did not differ in between the latter two (all p>.05). There was neither a main effect of the factor “group” nor any other significant interactions.

### Mental rotation speed

The univariate analysis of variance showed a significant main effect for “stimulus type”, *F*(2,76)  = 28.16, *p*<.01, *η^2^* = .426, but not for “group”, *F*(1,38)  = .12, *n.s*. The mental rotation speed did not differ significantly between body postures (*M* = 234.56°/s, *SD* = 116.68) and human figures, (*M* = 193.93°/s, *SD* = 117.57), *F*(1,39)  = 3.32, *n.s*. Furthermore, the mental rotation speed for cube figures (*M* = 96.96°/s, *SD* = 66.79) was slower than for human figures, *F*(1,39)  = 27.48, *p*<.001, *η^2^* = .413 and body postures, *F*(1,39)  = 87.58, *p*<.001, *η^2^* = .692. There was also no significant interaction between these factors, *F*(1,38)  = 1.92, *n.s*.

### Mental Rotation Intercept

**Figure 4:**
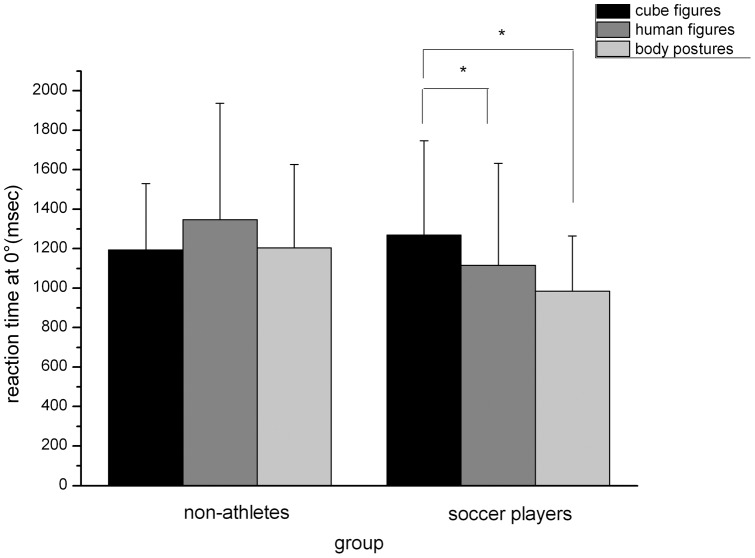
Reaction time at an angular disparity of 0° dependent on stimulus type and angular disparity (mean, standard deviation).

Analyzing the reaction time at an angular disparity of 0°, the univariate analysis of variance showed a main effect of “stimulus type”, *F*(2,76)  = 3.89, *p<*.05, *η^2^* = .093, and no effect for “group”, *F*(1,38)  = .98, *n.s*., but did show a significant interaction between these factors, *F*(2,76)  = 4.675, *p*<.05, *η^2^* = .110 (see Figure 4). This interaction is due to the fact that the reaction time at an angular disparity of 0° did not differ for non-athletes, *F*(2,38)  = 2.15, *p*<.05., but did differ for soccer players, *F*(2,38)  = 6.74, *p<*.01, *η^2^* = .26. For the soccer player group the reaction time for human figures and body postures was lower than that for cube figures, *F*(1,19)  = 4.76, *p<*.05, *η^2^* = .20 and *F*(1,19)  = 12.84, *p<*.01, *η^2^* = .40. The reaction time at 0° did not differ for this group between the human figures and the body postures, *F*(1,19)  = 2.51, *n.s*.

## Discussion

First of all this study shows a faster reaction time for embodied stimuli in soccer players compared to non-athletes. This effect could not be shown by analyzing error rates across angular disparities or mental rotation speeds but was evident when analyzing the reaction time for the angular disparity of 0°. The reaction time for 0° trials reflects the perceiving or encoding processes(stage 1–3, see introduction) and decision making processes (stage 6–7, see introduction) [17]. This means that even though the soccer player showed a faster reaction time of mental rotation for embodied figures (table 1), this result could not be attributed to the rotation process itself, because mental rotation speed did not differ between groups. For this, this faster reaction time might be due to a better ability to perceive or encode the objects or as part of the decision process in the form of a motor response by pressing the mouse button. To investigate which of the three aspects is relevant further studies should analyze the performance of athletes and non-athletes in visual comparison tasks and motor response tasks. Visual comparison time can be considered as a relevant component of the reaction time gain of the soccer players [26]: A faster reaction time could be revealed in eye-hand and eye-foot visual reaction times of young soccer players compared to young male non-soccer players. This is supported by the fact that advanced perceptual skills are characteristics of successful soccer players [27]. Results of another study suggest that athletes showed a faster simple reaction time in a computer-based test than non-athletes, which can be attributed to a faster processing speed [28]. This advantage in simple reaction time was also associated with performance in an everyday task, a street crossing-task. These studies are in line with the recently published results that soccer players showed a significant better measurement of executive functioning [4].

The advantage of soccer players compared to non-athletes was visible in reaction times but not in error rates. Within the analysis of error rates, the use of strategies could be analyzed. The use of a piecemeal strategy might be seen by a higher level of degradation of response accuracy as a function of angular disparity when compared to the use of a holistic strategy [17]. Our results show a higher level of degradation of response accuracy for cube figures than for embodied stimuli as a function of angular disparity (see Figure 3). This result shows that both soccer players and non-athletes use a more holistic strategy for embodied stimuli. However, since there was no significant 2-way interaction between group and angular disparity nor a 3-way interaction between group, angular disparity, and stimulus type in the degradation of response accuracy, our data suggest no difference in the strategy used between soccer players and non-athletes. The reaction time advantage might be an advantage of perception or decision but not an advantage of a different strategy use. To supplement these findings, eye-tracking could be used to clarify the strategies used by motor experts and non-motor experts [29].

The results of this study are in some ways contradictory to the study of Steggemann et al. [18] because an advantage of athletes compared to non-athletes in an object mental rotation task with embodied stimuli was shown. Both studies differ in several aspects such as the use of the objects figures (cubed vs embodied stimuli) as well as the different groups of athletes who have participated (rotational movement experts vs soccer players). Despite the differences, this study suggests that even in a same-different mental rotation task, which induces object transformations, soccer players show faster reaction times. It seems to be more interesting to ask which component of the mental rotation task in a same-different judgment is influenced by athletes with long time physical activityexpertise. In this study with soccer players it was shown that the influence was not due to the rotation process itself which also gives a hint that not the (possible) better executive functions or working memory processes contribute to better mental rotation performance of the soccer players. Instead this could be attributed to the encoding or perceiving process, or the motor response in the mental rotation task. To investigate this in further detail more studies must follow which use stimulus types comparable to the study of Amorim et al. [17] with different types of athletes. For example, gymnasts have extensive experience in turning around all three body-axes and display a more egocentric point of view [18]. Compared to this, soccer players are trained to use egocentric skills but also must use the exocentric perspective since they have to take into account their own body, the ball as the object, the bodies of the teammates, the bodies of the adversary team, and the soccer field. To supplement this experiment another study might be conducted with the between subject factor group (gymnasts vs. e.g. soccer players vs. non-athletes) and with cube figures, human figures, and body posture figures as stimuli types. In addition, the type of judgment must be varied (same-different vs. left-right judgments). Furthermore, gender might be an important factor due to the well-known gender differences in mental rotation performance [25], [8]. This is a factor, which has been neglected up to now in the investigation of motor effects in mental rotation and was also a limitation in this study.

In addition to the results of soccer players vs. non-athletes, the results show that mental rotation tasks using rotated cube figures needed more time to be solved than rotated embodied figures. This finding is also expressed by the slower mental rotation speed for cube figures. Furthermore, participants made more errors solving mental rotation tasks with cube figures, but only for figures which had an angular disparity of 90° or higher.

The poor performance on mental rotation tasks with cube figures in comparison with embodied figures is in line with a former study [17]. In this study faster reaction times were attributed to both body postures and human postures when compared to cube figures, however the body postures elicited slower reaction times than the human postures by a factor of four. We did not find this effect in our study. This difference might be due to the different experimental designs. In the former study this result was obtained by comparing the reaction times between human figures and body postures across experiments while we used a within subject paradigm. This design might have led to a “training” of body postures by presenting cube figures and human figures as well as the combination of this in the form of body postures in the same experiment. Figure 2 shows that the typical mental rotation time function could be seen with the mental rotation of cube figures but that it is not as prominent with embodied stimuli. This can be seen in analogy to the RTs of imagined perspective or egocentric transformations, which are often independent of angular disparity [30]. Egocentric transformations require the rotation of the viewer's reference frame relative to a stable allocentric frame [31]. Due to the small increases in reaction time dependent on increasing angular disparity with embodied stimuli in this study, one might conclude that within a same-different task using embodied stimuli, egocentric transformations “meaning transformations in respect to the one's own body do play a role.

This study has added to the literature of the coupling of perception and action and deserves further attention in the literature on embodied cognition. In a broader sense embodied cognition means that cognitive processes are rooted in the body and its interaction with the world [32] or that cognitive processes can only be understood when taking body representations into account. This study has shown that this relationship must be investigated in more detail. A differentiation is needed since mental rotation is composed of the three different cognitive main stages: a) the perceptual process, the identification and discrimination of the stimuli and the orientation, b) the mental rotation process itself and the judgment of parity, and c) the response selection and execution [13], [14]. Further studies should strive to contribute to the perception-action as well as the embodied cognition literature.
